# Development of Biopolymer Polylactic Acid–Cellulose Acetate–Silicon Dioxide Nanocomposite Membranes for Multifunctional Protective Textiles

**DOI:** 10.3390/polym17162237

**Published:** 2025-08-17

**Authors:** Irfan Farooq, Abdulhamid Al-Abduljabbar, Ibrahim A. Alnaser

**Affiliations:** Department of Mechanical Engineering, King Saud University, Riyadh 11421, Saudi Arabia; 443107001@student.ksu.edu.sa (I.F.); ianaser@ksu.edu.sa (I.A.A.)

**Keywords:** multifunctional textile, biopolymer membrane, nanocomposite, electrospun nanofibers, polylactic acid, acetic acid, silica nanoparticles

## Abstract

In this study, multifunctional nanocomposite membranes were fabricated using biopolymeric polylactic acid (PLA) and cellulose acetate (CA) composites via electrospinning. The hydrophobic nanocomposite membranes were reinforced with varying concentrations of silicon dioxide (silica/SiO_2_) nanoparticles. The developed PLA–CA–SiO_2_ nanofibrous membranes are characterized using field emission scanning electron microscopy (FE- energy-dispersive SEM), energy-dispersive X-ray (EDX), elemental mapping, X-ray diffraction analysis (XRD), Fourier-transform infrared spectroscopy (FT–IR), thermal gravimetric analysis (TGA), and differential scanning calorimetry (DSC) techniques. Various physical and mechanical properties of the bio-nanocomposite membrane, such as tensile testing, infrared thermal imaging, ultraviolet–visible spectroscopy (UV–Vis), water contact angle, hydrostatic pressure resistance, and breathability are also investigated. The analysis revealed that a small concentration of silica nanoparticles improves the morphological, mechanical, and thermal characteristics of nanocomposite membranes. The addition of silica nanoparticles improves the UV (A & B), visible and infrared blocking efficiency while also enhancing the waterproofness of protective textiles. The PLA–CA–SiO_2_ biopolymer nanocomposite membrane has a fibrous microstructure and demonstrated the tensile strength of 11.2 MPa, a Young’s modulus of 329 MPa, an elongation at break of 98.5%, a hydrostatic pressure resistance of 27 kPa, and a water contact angle of 143.7°. The developed electrospun composite membranes with improved properties provide strong potential to replace petroleum-based membranes with biopolymer-based alternatives, promising improved and wider usage for bio-related applications.

## 1. Introduction

Nanotechnology has sparked significant interest among researchers in developing multifunctional, sustainable, and technical textiles. One major function of such textiles is to provide protection against ultraviolet radiation, rain, water, chemical, and biological hazards. These textiles have applications in various sectors such as military, membrane distillation, sports, smart garments, healthcare, construction, and agriculture workers [1,2]. Human skin serves as the body’s primary barrier against external elements, maintaining physiological comfort by regulating body heat, blocking water droplets, and allowing the vapors (sweat) to escape. Inspired by human skin and plant leaves, microporous, breathable, and waterproof textiles are of significant interest for maintaining a dry and comfortable environment in harsh climate areas and extreme conditions [3,4]. In daily life, moderate exposure to sunlight is necessary for health; however, prolonged exposure increases the risk of permanent skin damage from ultraviolet rays, including sunburn, premature ageing, DNA damage, and skin cancer [3]. Protective textiles should exhibit adequate mechanical strength, thermal stability, waterproofness, and breathability. Nonwoven textiles are commonly categorized as either hydrophobic or hydrophilic membranes [5]. Hydrophobic membranes offer excellent waterproofness and breathability due to their microporous structure of fibers, where pore sizes are larger than water vapor molecules but smaller than the finest rain droplets. The rate of water vapor transmission in hydrophobic porous membranes is higher than in hydrophilic membranes; therefore, they exhibit good moisture and air permeability and have attracted considerable research interest in the development of protective textiles. They are lightweight, cost-effective, offer excellent protection, and exhibit a high level of comfort for end users. The properties of these micro/nanoporous nonwoven membranes can be tailored through pre- or post-treatment processes, such as polymer blending, nanoparticle incorporation, surface energy reduction, pore size and porosity modification, and heat treatment [6].

Currently, microporous membranes fabricated of poly tetrafluoroethylene (PTFE), polyether sulfone (PES), polyvinyl chloride (PVC), and polyvinylidene fluoride (PVDF), etc., are commercially utilized as waterproof and breathable protective textiles because of their mechanical strength, thermal properties, protection, and comfort. These synthetic polymeric petroleum-based membranes have some constraints regarding elasticity, environmental, and public health concerns due to the employment of fluorinated compounds to lower the surface energy and utilization of toxic chemicals such as perfluorooctanoic acid (PFOA) and perfluoro-octane-sulfonate (PFOS) during their synthesis process. The development of environmentally friendly and elastic microporous membranes with appropriate mechanical strength and thermal stability, waterproofness, and breathability characteristics is challenging [2,6,7].

Hydrophobic characteristics can be introduced in microporous nonwoven membranes by lowering the surface energy or developing the rough structure on the surface of membranes. Commonly, fibrous membranes are modified with low-surface-energy chemicals, the addition of nanoparticles, or by coating treatments. Previously, fluoropolymers were extensively used to reduce the surface energy of membranes and to enhance hydrophobicity, but they have an adverse impact on the environment [8,9]. There are different techniques which can generate surfaces with hydrophobic characteristics, such as phase separation, biaxial stretching, template synthesis, melt blowing, wet coagulation, the sol–gel method, and electrospinning. However, these techniques have some limitations regarding processing the variety of polymers, complexity in processing, tailoring the morphology of microstructure, high cost, or difficulty in regulating breathability and waterproofness simultaneously [1,10,11].

Electrospinning is one of the most attractive techniques utilized to fabricate continuous and uniform micro/nanoporous membranes of varied compositions. It has the ability to produce tunable fiber diameters with small pore size, high porosity, high specific surface area, and interconnected and controllable porous structures. Fibrous membranes can be modified easily to enhance the specific properties of mats, such as mechanical, thermal, electrical, and optical characteristics [5,6,12]. In the electrospinning process, properties of polymer nanofibers are tuned by manipulating the environmental, solution and process parameters [13]. According to the Cassie and Baxter Model, changes in surface roughness and porosity of electrospun fibers alter the surface energy, which results in the change of wettability of membranes [14]. The microporous structure of electrospun mats facilitates moisture, vapor transmission, provides water and wind resistance so that they can be used for high-performance waterproof and breathable membranes. Generally, various nanoparticles, such as titanium dioxide, silicon dioxide, zinc oxide, silver, gold, and graphene, etc., are incorporated into electrospun membranes, which contribute to ultraviolet, microbial, and chemical protection and also enhance the mechanical strength, thermal insulation, and hydrophobic characteristics. Different types of electrospinning processes are utilized to generate these textures, such as single-jet or multi-jet electrospinning, melt electrospinning, and coaxial electrospinning [1,13].

The community of membrane technology is concerned about the detrimental impacts of petroleum-based polymers on the environment and public health. In 2019, the European Bioplastics and the Nova-Institute published their general recommendations to encourage the employment of sustainable products by promoting the development and usage of bio-based materials. This recognition boosted the research development in biopolymers. Recently, biopolymers such as poly (ethylene glycol) (PEG), poly (lactic acid) (PLA), chitosan, cellulose derivatives, etc., have attracted research attention in academia and industry due to their sustainability, biodegradability, and biocompatibility characteristics [11,15,16].

Polylactic acid is a biodegradable and sustainable thermoplastic material and a potential candidate to replace petroleum-based polymers. It is an aliphatic polyester with sustainability, biodegradability, low cost, spinnability, a low carbon footprint, reasonable mechanical and thermal properties, ultraviolet resistance, and antimicrobial activity derived from natural resources [16,17,18]. PLA nanofibrous mats have been utilized in numerous applications such as drug-controlled release [19], scaffolds [20], tissue engineering [21], water treatment [22], food packaging [23], filtration masks [24], and agricultural products, etc. However, there are certain parameters which hinder its commercial utilization, such as brittleness, low tensile strength, low toughness, less elongation at break, and low thermal stability. Different techniques have been applied to further improve its properties, such as polymer blending [18], copolymerization [25], crosslinking [26], etc. Cellulose acetate (CA) is also a biodegradable thermoplastic material that has tunable mechanical properties. It is utilized as an additive polymer in composite materials to improve tensile strength and toughness, reduce vulnerability to brittleness, and enhance thermal stability [27,28,29].

The properties of composite materials are highly influenced by the interaction between additive components. It is reported that the incorporation of nanosized fillers in a polymer matrix increases the filler–matrix interface and enhances the properties of composite materials at low concentrations [30,31]. Hao et al. developed multifunctional PDMS/SiO_2_@PLA textile with good breathability and super hydrophobicity via coaxial electrospinning [32]. Yang et al. fabricated phase change nonwoven fabric via 3D printing with enhanced thermal, radiation resistance properties, the multifunctional membrane has good hydrophobicity and breathability [33]. Gu et al. fabricated PLA-based hydrophobic/super hydrophilic Janus fibrous membranes via electrospinning combined with the sol–gel method. The Janus PLA(e–spun)/TiO_2_@PDA–PLA showed good mechanical properties, the directional moisture transport of vapors, and ultraviolet protection [3]. Lee developed a polypropylene nonwoven fibrous membrane by utilizing the electrospinning technique and analyzed the effect of zinc oxide (ZnO) nanoparticles on ultraviolet protection, moisture vapor transport, and thermal comfort characteristics at different web area densities of nanoparticles [34]. Ryu et al. modified the zinc oxide nanoparticles with hexadecyltrimethoxysilane (HDTMS) to enhance the hydrophobicity, UV protection, and dispersibility of nanoparticles and compared it with neat electrospun polylactide [35]. Zhao et al. fabricated a waterproof and breathable membrane of poly (styrene-b-butadiene-b-styrene) (SBS) by using the electrospinning technique. The SBS/PDA/SiO_2_ membrane showed super hydrophobicity, enhancement in resistance to hydrostatic pressure, and demonstrated water vapor transmission and air permeability [36].

In previous work, electrospun composite membranes of PLA-CA were developed with different concentrations of CA and examined to figure out the optimum concentration that results in most favorable behavior [37]. This included mechanical and thermal properties, hydrophobicity, and hydrostatic pressure resistance; the result of this revealed that the composite with concentration PLA 95–CA-5% is the best combination. Encouraged by this result, and by the positive effect of the introduction of ZnO and SiO_2_ in enhancing other types of composite nanomembranes [34,35,36], the goal of this work is to combine SiO_2_ into the PLA-CA composite nanomembrane to develop and characterize a sustainable biodegradable biopolymer PLA–CA–SiO_2_. Different concentrations of the SiO_2_ in the composite will be examined to identify the best combination. The resulting PLA–CA–SiO_2_ composite is hoped to possess favorable characteristics comparable to those of petroleum-based polymers, to enhance the utilization of biopolymers in different scientific and commercial applications, and to reduce environmental pollution resulting from petroleum-based polymer waste.

## 2. Materials and Methods

The base constituents of nanocomposite membranes are polylactic acid (PLA, LX175^®^, Filabot, Barre, VT, USA) and cellulose acetate (CA), which were acquired from Sigma-Aldrich (St. Louis, MO, USA). Silicon dioxide nanoparticles were purchased from Sigma-Aldrich. Solvents dichloromethane (DCM), N,N-dimethylformamide (DMF), acetone (≥99.5%), and acetic acid, utilized to prepare the solution for the electrospinning process, were purchased from Sigma-Aldrich. Deionized water was used to measure the water contact angle. The electrospinning machine NF-500, MECC (from Fukuoka, Japan) was used to process the composite solution.

**Figure 1:**
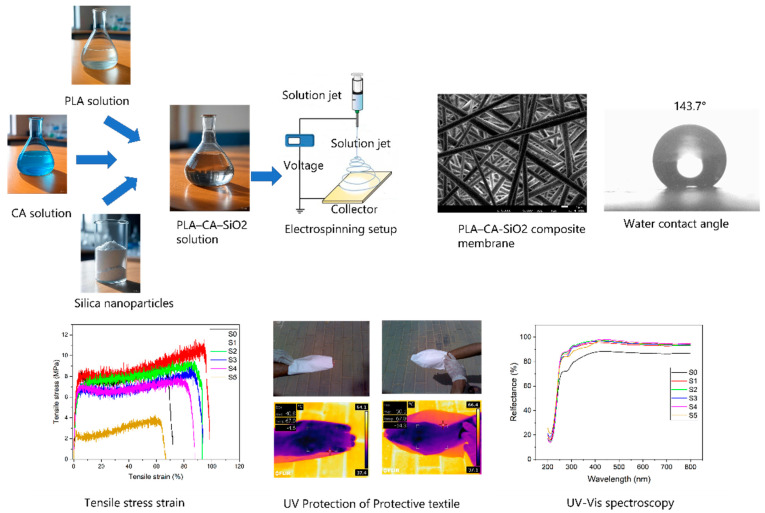
Schematic workflow diagram used to prepare PLA–CA–SiO_2_ composite membranes.

First, a 10 wt.% concentration of PLA was dissolved in DMF and DCM solvents with a 1:4 volume mixture ratio, respectively; the solution was magnetically stirred for three hours and kept at 40 °C and then cooled to ambient temperature. Similarly, a 17 wt.% concentration of CA was dissolved in acetic acid and acetone solvents with a 1:3 volume ratio, respectively, magnetically stirred for six hours and heated at 45 °C, and then cooled to room temperature. The composite solution was prepared by mixing CA 5% and PLA 95% solutions, as discussed in [37]. Afterwards, silica nanoparticles were blended in a PLA 95–CA 5 composite solution at different concentrations. The blend solution was prepared with 0.5, 1.5, 3, 5, and 7% (*w*/*w*) concentrations of silica nanoparticles to examine the effect of variable concentration. The solutions are kept on a magnetic stirrer overnight at room temperature to reach homogeneous status. The composite solution was filled in a 10 mL syringe having needle diameter of 0.6 mm, at the feed rate of 0.8 mL/h, with tip-to-collector distance kept at 15 cm, and the processing of the solution is performed at an applied voltage of 18 kV. A schematic workflow diagram of the preparation process of PLA–CA–SiO_2_ composite membranes is shown in Figure 1.

Resulting composite membranes of different concentrations of silica nanoparticles were labelled as S0, S1, S2, S3, S4, and S5, referring to 0, 0.5, 1.5, 3, 5, and 7% silica concentrations, respectively, as shown in Table 1.

### Characterization and Measurements

Morphological characteristics of biopolymer PLA–CA–SiO_2_ composite mats were investigated with a field emission scanning electron microscope (FE-SEM: JSM-7600, JEOL, Tokyo, Japan). A small piece of composite membrane was cut and fixed on a stub with carbon tape for further processing. The specimen was coated with platinum to increase their conductivity. FE-SEM analysis was performed under high vacuum conditions, and the fibrous microstructure of composite membranes was analyzed at the magnification of 5000×. EDX analysis was performed to determine the composition of nanocomposite membranes. To determine the dispersion of silica nanoparticles in composite membranes, elemental mapping analysis was also performed. Average fiber diameter was measured with ImageJ 1.54j software.

Mechanical properties of composite membranes were evaluated with Instron apparatus (ElectroPuls, Bucks, UK). In tensile testing, the gauge length of composite mats was kept at 20 × 45 mm, and the experimentation was performed at a crosshead speed of 5 mm/min. First, the membrane specimen was adjusted in a paper frame for easy handling and adjustment of the sample in the machine’s hydraulic grips. After the sample was gripped in the machine, the paper frame was cut with scissors. Tensile strength, Young’s modulus, and elongation at break were calculated from tensile stress–strain curves. Thermal characteristics of composite membranes were investigated with thermal gravimetric analysis (TGA) and differential scanning calorimetry (DSC) equipment (Q600, TA Inc., New Castle, DE, USA). A membrane sample of 10 to 12 mg weight was placed in a ceramic pan and the machine operated under ramp conditions with the temperature range of 25 to 600 °C at the heating rate of 10 °C min^−1^. The experiment was operated under a nitrogen gas environment.

The crystallinity of multifunctional composite membranes was investigated by X-ray diffraction analysis (XRD–7000, Shimadzu, Kyoto, Japan). Specimens were processed at a continuous scanning rate of 2°/min, and a 2θ degree range is applied from 5° to 90°. Functional groups attached to the composite membranes were determined with Fourier-transform infrared spectroscopy (FT–IR), and the composite mats were scanned in a range of 500 to 4000 cm^−1^. Reflection and absorbance of UV-Vis radiations capability of composite membranes were evaluated with UV–Vis spectroscopy. A UV–Vis–NIR scanning spectrophotometer (UV3600, Shimadzu, Kyoto, Japan), with a wavelength range of 200 to 800 nm, was used to measure optical properties of composite membranes. The infrared blocking efficiency of composite membranes was analyzed with an IR thermal camera (FLIR, E64501, Wilsonville, OR, USA). The visual and thermal images were captured of a human hand covered with protective textiles under direct sunlight. In thermal images, box max temperature is used as the current temperature of protective textiles.

The hydrophobicity of composite membranes was determined with a CA goniometer (OCA 15EC, Data Physics, Riverside, CA, USA). The protective textiles were also tested for breathability and hydrostatic pressure resistance. The breathability and hydrostatic pressure resistance of composite mats were determined by manual setup. For breathability, multifunctional composite textile was clamped in a filtration assembly flask moderately filled with tap water, and compressed air passed through it. The breathability of mats was analyzed by observing the air bubbles coming out from water. The waterproofness of composite mats was evaluated by measuring hydrostatic pressure resistance; the setup was manually developed in the laboratory, as reported in [38]. Composite membranes were adjusted in the filtration assembly partially filled with tap water; through a vacuum pump, suction pressure was developed. The pressure on composite membrane was regulated with a change in vacuum suction pressure. Vacuum pressure was increased gradually until water droplets appeared at the bottom side of the composite mats.

## 3. Results and Discussion

### 3.1. Morphological Analysis

**Figure 2:**
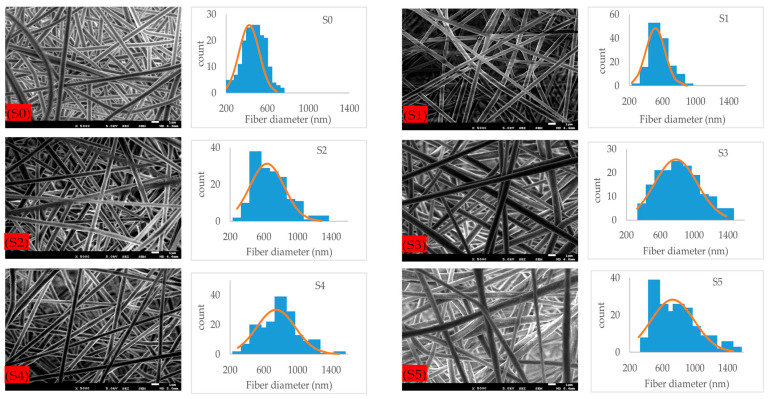
FE-SEM microstructural figures and normal distribution of PLA–CA composite membrane fibers at different concentrations of silicon dioxide nanoparticles.

**Figure 3:**
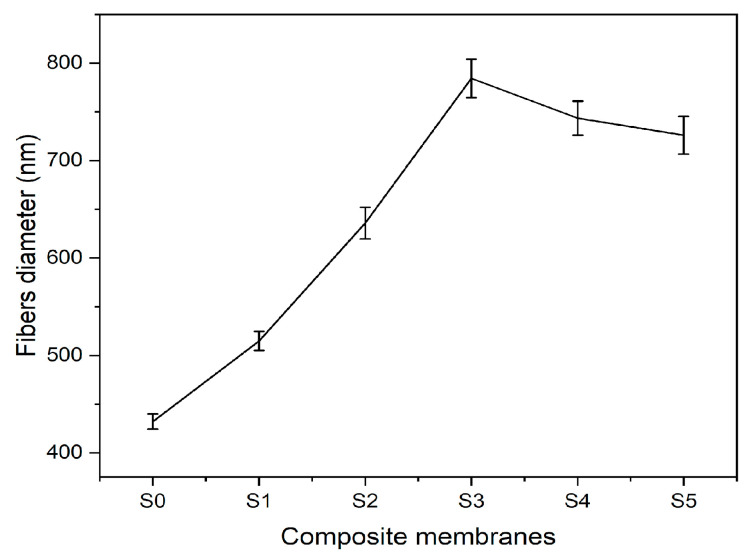
Average fibers diameter of PLA–CA–SiO_2_ composite membrane.

Microstructural images of fibrous composite membranes and the normal distribution of fibers diameters of electrospun biopolymer composite textiles are shown in Figure 2. SEM images represent smooth, homogeneous, continuous, and beads free fibers. The electrospun composite membrane sample with no SiO_2_ content (sample S0) has an average fiber diameter of 432 ± 7.8 nm with a standard error. The sample with SiO_2_ content 0.5% (sample S1) has an average fiber diameter of 514 ± 9.8 nm, which is around a 20% increase compared to the pure composite. Further increments in the concentration of silica nanoparticles to 1.5% (sample S2) and 3% (sample S3) produce consistent increases in the average fiber diameter rises to 635 ± 16.2 nm and 784 ± 19.8 nm, respectively, reaching a maximum increase over 70% over the original average diameter. Individual charts for each sample are shown in Figure 3. Up to this point, the average diameter of composite fibers increases consistently as silica nanoparticles concentration increases, where such nanoparticles act as nucleation sites for the crystallization in PLA–CA–SiO_2_ composite and enhance the interfacial adhesion between the PLA–CA matrix composite and the fibers. However, a further increase in the silica nanoparticles concentration in the composite solution slightly decreases the average fiber diameter. The composite samples S4 and S5 have average fiber diameters of 744 ± 17.4 nm and 725 ± 19.3 nm, respectively. Such a benign reduction in average fiber diameter may be attributed to repulsive forces during the electrospinning process among silica nanoparticles due to an increase in conductivity, which may decrease the entanglement of composite polymer chains.

**Figure 4:**
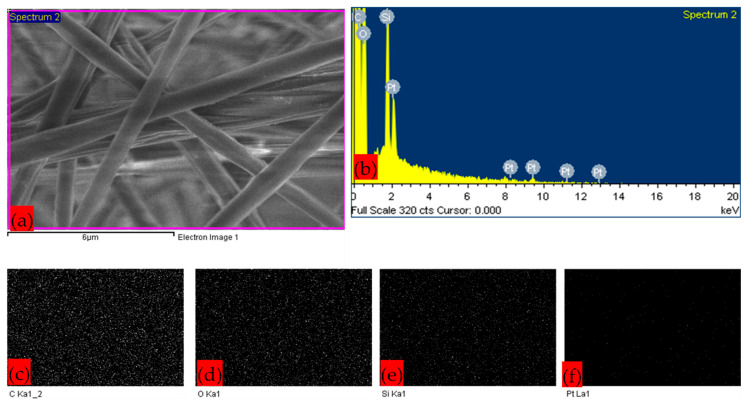
SEM–EDX figure of S3 PLA–CA–SiO_2_ composite membrane, with 10,000× magnification: (**a**) SEM; (**b**) EDX; (**c**) elemental mapping of carbon element; (**d**) elemental mapping of oxygen element; (**e**) elemental mapping of silicon element; (**f**) elemental mapping of platinum element.

Figure 4 represents the EDX and elemental mapping images of the composite membrane sample S3, which has the highest fiber average diameter. The images confirm the presence of silica nanoparticles. Carbon and oxygen are the building blocks of polymer composite. The platinum element was coated to enhance the conductivity of the membrane sample for processing in SEM. Overall, PLA–CA–SiO_2_ composite membrane samples S1–S5 exhibited comparable morphological nanofibrous architecture and nanoparticle characterization.

### 3.2. Thermal Analysis

**Figure 5:**
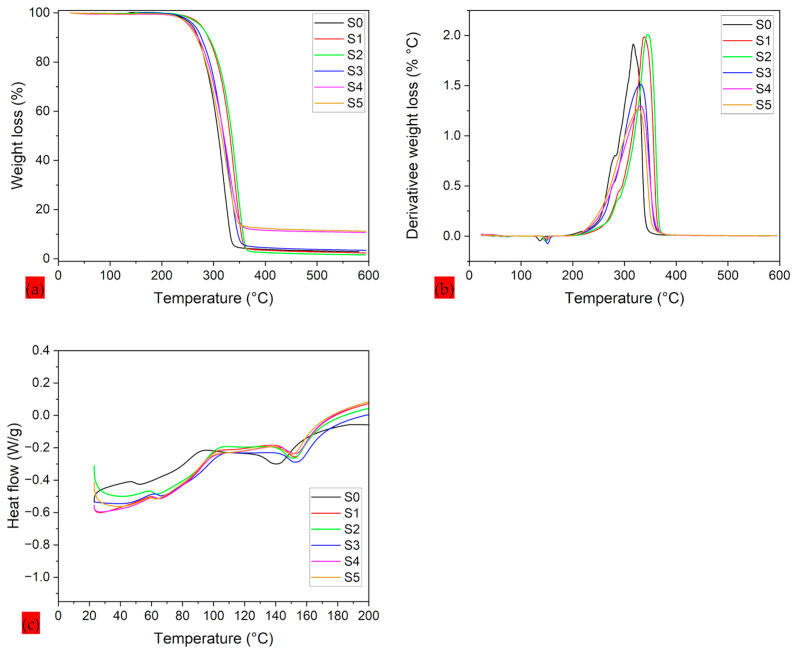
Thermograms of PLA–CA–SiO_2_ composite membranes: (**a**) TGA thermograms; (**b**) DTG thermograms; (**c**) DSC thermograms.

A comprehensive thermal analysis of PLA–CA–SiO_2_ electrospun composite membranes is shown in Figure 5. Thermal degradation and threshold temperatures are important parameters used to optimize composite systems [39]. A TGA thermogram is used to analyze onset temperature and the temperature at different weight loss percentages of composite. The differential thermogravimetry (DTG) curves are used to measure the maximum peak temperature of weight loss curves, and DSC curves are utilized to measure glass transition ($T_{g}$) and melting temperatures ${(T}_{m})$ of biopolymer composite mats. TGA thermograms represent a single degradation step, and the main weight loss of the composite polymer was observed between 250 °C and 350 °C. Up to 100 °C, no thermal dehydration was observed, which confirmed the hydrophobic nature of protective membranes. S0 composite membrane demonstrated onset temperature ($T_{on-set}$), 50% ($T_{50\%\;WL}$) weight loss, and peak maximum temperature ($T_{d-max}$), $T_{g}$, and $T_{m}$ temperatures of 298.9, 322.27, 329.65, 61.31, and 144.24 °C, respectively. Further increasing the concentration of silica nanoparticles to 1.5% (S2 Samples) results in increasing the thermal stability of composite membranes. The S2 composite membrane has shown the maximum thermal stability and demonstrated the $T_{on-set}$, $T_{50\%\;WL}$, $T_{d-max}$, $T_{g}$, and $T_{m}$ temperatures of 313.6, 337.1, 345.6, 59.23, and 153.5 °C, respectively. Further enhancing silica concentration to 3% to 7%, the thermal properties of composite membranes decrease. The thermal properties of composite membranes are summarized in Table 2. The thermographs of PLA–CA–SiO_2_ composite membranes are consistent with those reported in the literature [40]. In PLA–CA–SiO_2_ composite membranes, the degree of crystallinity is proportional to thermal stability. The TGA thermogram curves for S1 and S2 composite membranes are at higher temperatures, indicating higher thermal stability and the crystallinity of the membranes. Further increases in the silica concentration thermograms produce TGA curves with lower temperatures, which could be attributed to aggregations of silica nanoparticles during the electrospinning process, reducing the chain mobility which leads to decreases in thermal stability and crystallinity [41]. By increasing the concentration of silica nanoparticles, the percentage of the residue mass increases. S1 composite has a residue mass of 2.3%, while S5 membrane has a residue mass of 11.2%. As in the analysis for the average fiber diameter, the optimum value of the property is found at an intermediate range of silica concentration.

### 3.3. X-Ray Diffraction (XRD)

**Figure 6:**
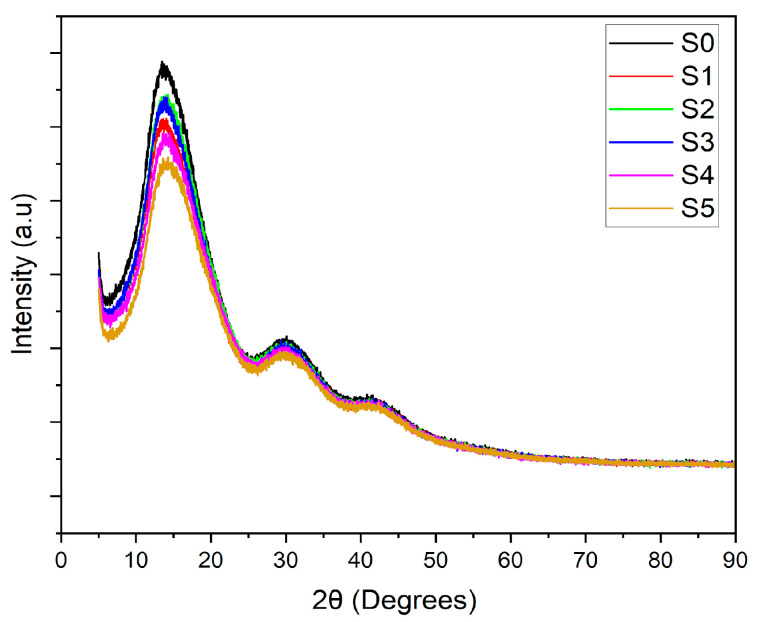
XRD curves of PLA–CA–SiO_2_ composite membranes.

The crystallinity of the PLA–CA–SiO_2_ composite membranes is investigated with X-ray diffractometry curves shown in Figure 6. The PLA-CA composite membrane demonstrated a semicrystalline curve showing a peak at Bragg’s angle (2θ) of 13.8 degrees and broader peaks at Bragg’s angle of 30 and 42 degrees. By blending silica nanoparticles, the intensity of the curves decreases, as also reported in [42], all composite membranes demonstrated the peaks at the same Bragg’s angle. The intensity of XRD curves increases by increasing the concentration of silica nanoparticles from 0.5 to 3% concentration; further increases in concentration reduce the intensity of peaks. The decrease in intensity could be ascribed to the agglomeration of silica nanoparticles which reduce nucleation effect.

### 3.4. FT–IR Analysis

**Figure 7:**
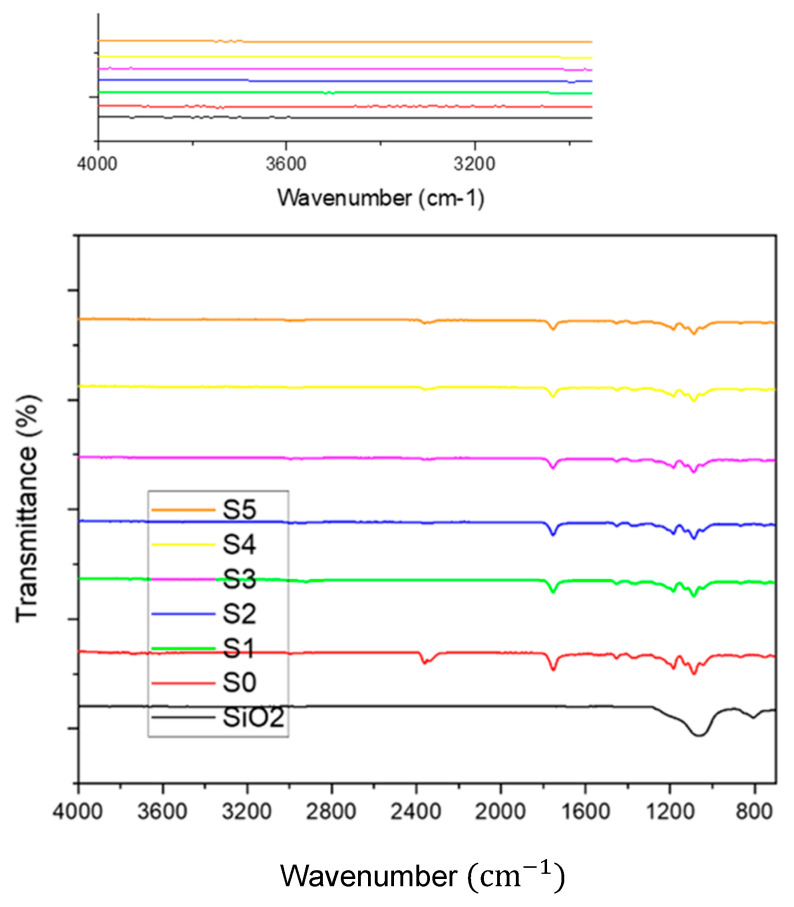
FT–IR spectrum PLA-CA-SiO_2_ composite membranes.

Figure 7 demonstrates FT–IR spectrum of silica nanoparticles, PLA–CA, and PLA–CA–SiO_2_ composite membranes. FT–IR spectrum of SiO_2_ powder has broad peaks around 810 cm^−1^ and 1068 cm^−1^ representing the symmetric stretching vibrations of the Si–O–Si bonds. FT–IR spectra of PLA-CA composites have absorption bands at 1184 cm^−1^ and 1751 cm^−1^, representing the C–O and carbonyl (C=O) stretching in ester groups. Peaks at 1087 and 2996 cm^−1^ are attributed to C–O and C–H stretching vibrations that represent both PLA and CA in the composite. Bands at 1380 and 1450 cm^−1^ demonstrate CH_3_ and CH_2_ bending vibrations of composite components. Small intensity absorption peaks from 3200 to approximately 4000 cm^−1^ demonstrate the hydroxyl (O–H) groups, indicating the hydrogen bonding in the PLA–CA composite [37]. The FT–IR spectra of PLA–CA–SiO_2_ electrospun composite membrane have absorption peaks around 1100 cm^−1^, associated with asymmetric stretching of Si–O–Si bonds from the silica components. The peak at 1754 cm^−1^ is likely associated with carbonyl (C=O) stretching vibrations in PLA–CA composite. The absorption peaks at 1380 cm^−1^ and 1450 cm^−1^ represent the CH_3_ and CH_2_ bending vibrations of the composite membrane. The addition of silica nanoparticles in the composite membrane introduces spectral features attributed to the Si–O–Si bonds, while in spectra, characteristic peaks related to PLA–CA are still represented.

### 3.5. Ultraviolet–Visible Spectroscopy

**Figure 8:**
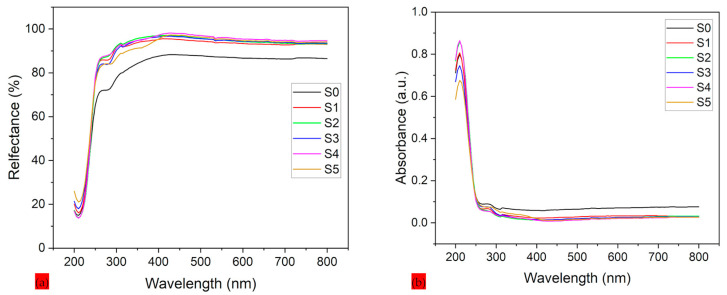
UV spectroscopy of PLA-CA-SiO_2_ composite membranes: (**a**) reflection spectroscopy; (**b**) absorbance spectroscopy.

Figure 8a,b showcase the reflection and absorption spectroscopy of PLA–CA–SiO_2_ electrospun composite membranes and the effect of silicon dioxide nanoparticles across the UV–Vis spectrum. For Figure 8a, the protective specimen without silica nanoparticles (S0) demonstrated lowest reflectance, especially in the UV region (200–400 nm), with values increasing steeply beyond 400 nm. This minimal UV reflection can be attributed to high absorption or scattering in PLA–CA composite membranes. All silica-reinforced composites from S1 to S5 exhibit increased reflection in VU range, with effect becoming more prominent at higher concentrations of silica nanoparticles. This improvement in protective textile can be attributed to the enhanced surface roughness and scattering centers generated by silica nanoparticles, which redirect UV photons away from protective textile rather than allowing them to absorb. Approximate stabilization in reflectance is observed above 400 nm across all protective textiles, which suggests the silica effect is most prominent in the UV region due to the distribution and size of silicon dioxide nanoparticles interacting more effectively with shorter wavelengths. The protective textile manages the heat of the body by evaporating the vapors and also helps to protect the skin from burning and aging and reduce the chances of cancer [43].

Figure 8b demonstrates the UV-Vis absorbance spectra of protective textiles. The S0 mat exhibits the highest absorbance, with a sharp peak in the UV region (200–300 nm), followed by sharp decline beyond 400 nm. This UV absorption in PLA–CA composite matrix can be attributed to electronic transitions within the polymer structure. The blending of silica nanoparticles results in notable reduction in UV absorbance. Silica nanoparticles act as blocking barriers or scattering agents that contribute to reduction in UV absorbance. For all protective textiles, absorbance remains minimal beyond 400 nm, which indicates that the silica effect is more prominent in the UV region.

**Figure 9:**
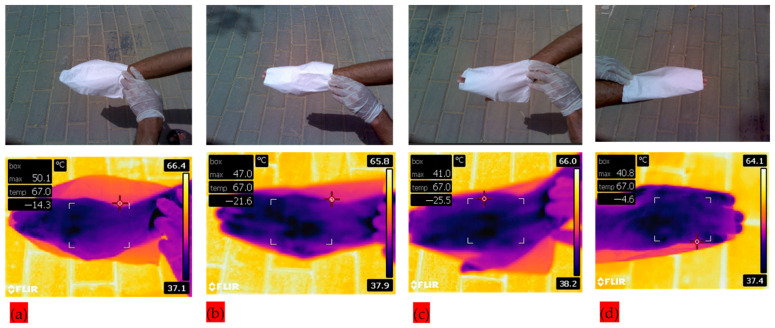
IR Spectroscopy of PLA–CA–SiO_2_ multifunctional textile; visual and thermal images of protective textile: (**a**) S1: 0.5% SiO_2_; (**b**) S2: 1.5% SiO_2_ protective textile (**c**) S3: 3% SiO_2_ (**d**) S4: 5% SiO_2_.

Additionally, infrared thermal images were captured to analyze the infrared blocking efficiency of PLA–CA–SiO_2_ composite membranes. Human skin was exposed under sunlight with protective textiles of different SiO_2_ concentrations in order to investigate the effect of introduction of silica nanoparticles, and temperature is measured by infrared camera in the premises of King Saud University, Riyadh, KSA. Figure 9 shows visual and infrared thermal images of the human hand covered with protective textile membranes. Cases S1–S4, represented by Figure 9a–d) in the graph, respectively. The measured temperature drops gradually as the silica content is increased from an initial value of 50.1 °C, for S1, as shown in Figure 9a, to a minimum value of 40.8 °C for S4, as shown in Figure 9d. The biopolymer developed protective textile with 5% silica resulted in a significant improvement over the one with only 0.5% silica, with a temperature reduction of more than 18% in the human hand. It is noted that there is very little difference between cases of to S3 in Figure 9c and S4 in Figure 9d. This is consistent with the result of the average fiber diameter of the two membranes as the average diameter reaches a peak for S3 and then starts to decrease slightly for S4. This result may have significance in applications for the protection of the human body from direct sunlight exposure.

### 3.6. Hydrophobic Analysis

**Figure 10:**
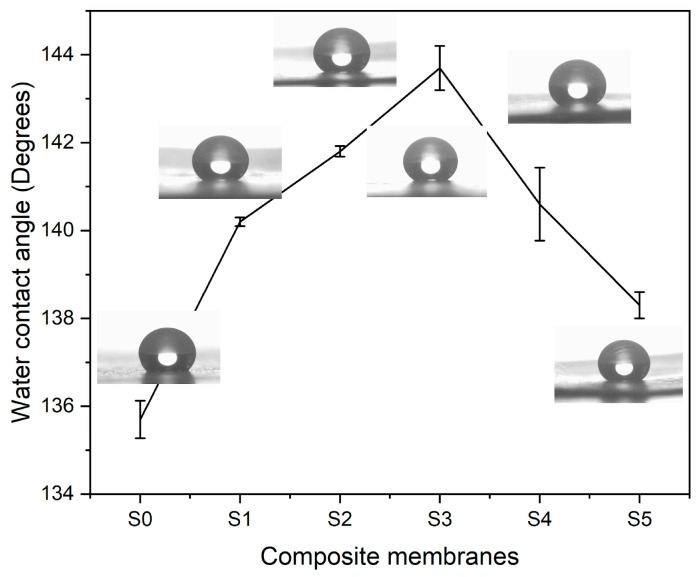
Water contact angle of PLA–CA–SiO_2_ composite membranes.

Water contact angle (WCA) observations were carried out to analyze the hydrophobic characteristics of electrospun composite textiles. Figure 10 shows WCA measurements of composite at various concentrations of silica nanoparticles. The PLA–CA composite membrane demonstrated the WCA of 135.7°. By increasing the concentration of silica nanoparticles, the WCA of composite membranes increases. The composite membranes S1, and S2 have water contact angles of 140.3° and 141.6°. S3 multifunctional membranes demonstrated a maximum WCA of 143.7°. An increase in WCA can be credited to an increase in surface roughness of composite membranes due to the presence of silica nanoparticles. These rough surfaces trap air that reduces contact area between water droplets and membrane surface. Further increases in the concentration of silica nanoparticles reduce the WCA. S4 and S5 membranes have WCA values of 140.3° and 138.3° respectively. At higher concentrations, due to the agglomeration of silica nanoparticles, WCA decreases [44,45].

### 3.7. Hydrostatic Pressure Resistance

**Figure 11:**
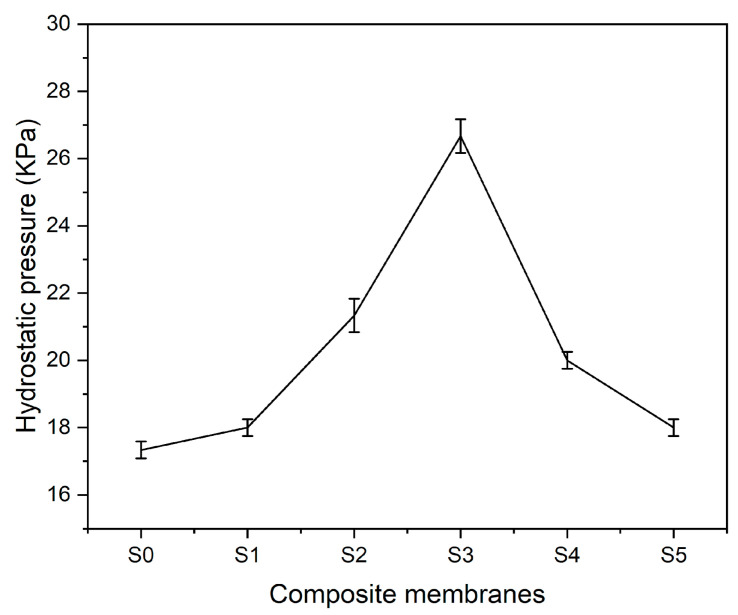
Hydrostatic pressure resistance of PLA–CA–SiO_2_ composite membranes.

The ability to resist hydrostatic pressure further verifies the waterproofness and strength of composite membranes. Hydrostatic pressure resistance setup is manually established in the lab. Hydrostatic pressure on the multifunctional textile surface is manipulated by the suction pressure of the vacuum pump. Pressure on the gauge is noted until drops of water become visible on the bottom surface of composite membranes. Figure 11 represents the characteristics of composite textiles in terms of resisting hydrostatic pressure. PLA–CA composite membrane shows the hydrostatic pressure resistance of 17.5 kPa. By enhancing the concentration of silica nanoparticles, hydrostatic pressure resistance increases. The S3 composite membrane has shown the maximum hydrostatic pressure resistance of 27 kPa. A similar trend was observed for hydrostatic pressure resistance in the literature [40]. This further increased silica concentration and decreased hydrostatic pressure resistance. The S5 composite membrane had a hydrostatic pressure resistance of 18 kPa.

**Figure 12:**
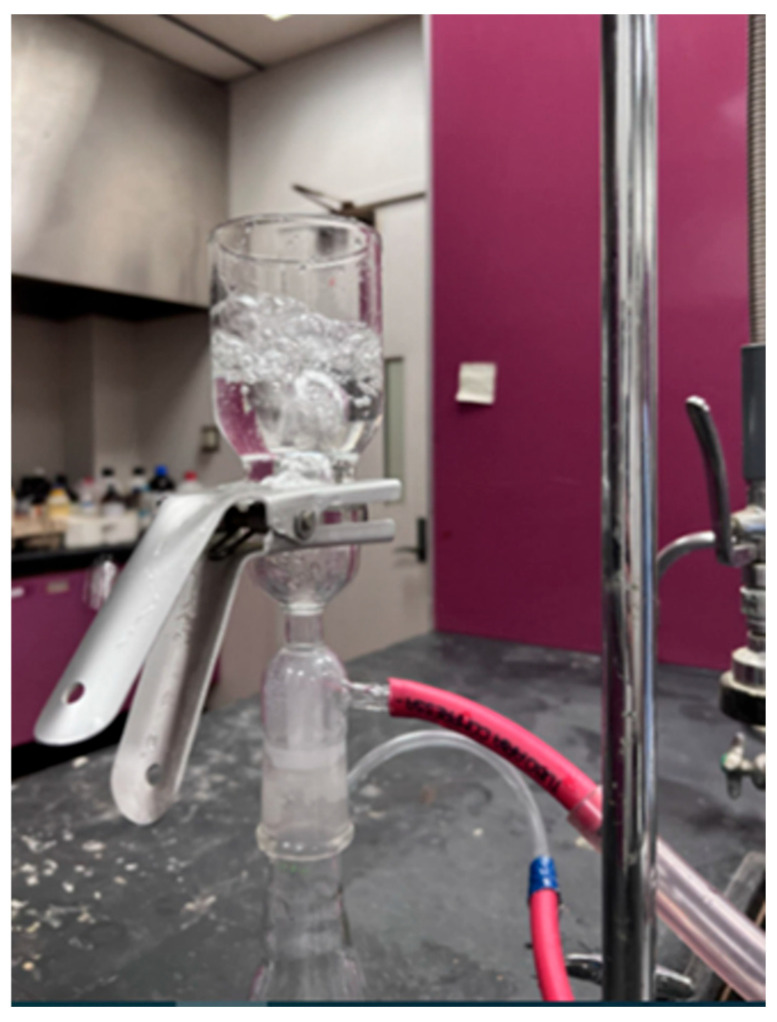
Breathability of PLA–CA–SiO_2_ composite membranes.

The effect of silica nanoparticles on air permeability was analyzed by a manually developed breathability apparatus. The manual setup is developed as reported in [38]; the membrane is adjusted in an assembly glass flask, and water is filled on its top surface. Compressed air is passed from the bottom side; as air passes through the membrane, air bubbles come out from the water, as shown in Figure 12. All composite membranes were tested for breathability, and they demonstrated air permeability.

### 3.8. Tensile Test

**Figure 13:**
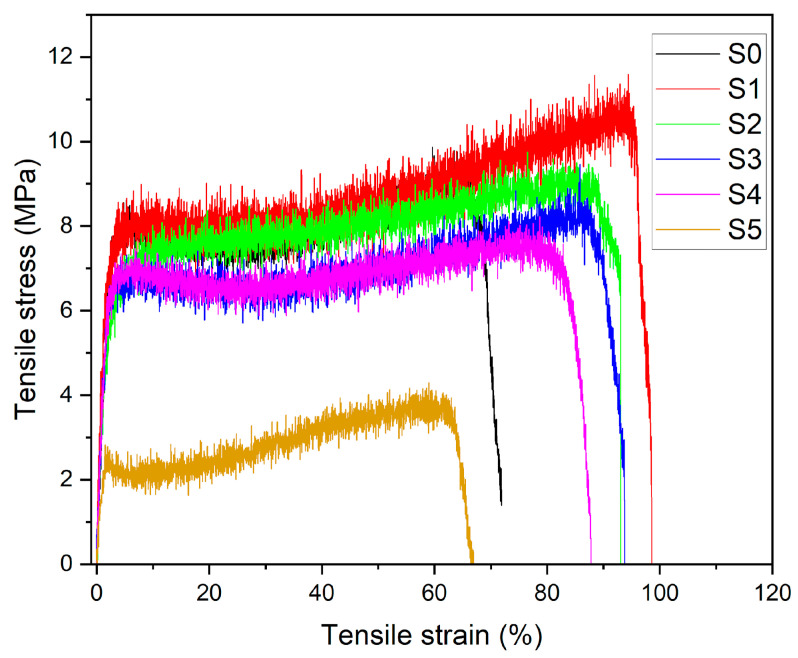
Tensile stress–strain curves of PLA–CA–SiO_2_ composite membranes.

The mechanical behavior of PLA–CA–SiO_2_ composite membranes was evaluated by tensile testing. The tensile test can be performed on either a single electrospun nano/microfiber or on a membrane [46,47]. Most researchers prefer to analyze the mechanical properties of electrospun membranes with a universal testing machine [48,49]. The tensile test demonstrates how material behaves in response to an axially applied load. Figure 13 shows the stress–strain curves of composite membranes. The PLA–CA composite membrane has a tensile strength of 9.3 MPa, a Young’s modulus of 291 MPa, and an elongation at break of 72.9%. Initially, the addition of silica nanoparticles enhanced the mechanical properties of composite membranes. The S1 membrane has demonstrated a maximum tensile strength of 11.2 MPa, a Young’s modulus of 329 MPa, and an elongation at break of 98.5%. Further increasing silica concentrations reduces mechanical properties. S2 membrane has a tensile strength of 9.7 MPa, a Young’s modulus of 320 MPa, and an elongation at break of 94%. S5 has a tensile strength of 3.9 MPa, a Young’s modulus of 112 MPa, and an elongation at break of 66%. At lower concentrations, silica nanoparticles enhance the interfacial adhesion and nanofibers morphology of composite membranes. However, at higher concentrations of silica, nanoparticles cause aggregation, which may hinder the chain mobility and disrupt the composite nanofibers structures. All such changes together lead to the reduction in mechanical properties of composite membranes [50,51].

## 4. Conclusions

Biopolymeric polylactic acid and cellulose acetate composite membranes, reinforced with silica nanoparticles at 0.5, 1.5, 3, 5, and 7% (*w*/*w*) concentrations, were successfully produced and analyzed for multifunctional protective textiles applications. The PLA–CA composites demonstrated good mechanical, thermal, hydrophobic and hydrostatic pressure resistance. Furthermore, these properties were enhanced by incorporating silica nanoparticles into PLA–CA composite membranes. All composite membranes exhibited good morphological characteristics. As the concentration of silica increased, the fiber diameters also increased, reaching a maximum of 784 nm in the S3 composite membrane. Beyond this concentration, further increases in silica content led to a reduction in fibers diameters. EDX and elemental mapping confirmed the presence of silica nanoparticles in composite membranes. The thermal properties of the composite improved the concentration of silica nanoparticles by up to 1.5%; beyond that, thermal stability decreased. XRD results confirmed a reduction in crystallinity with increasing silica concentrations. FT–IR analysis confirmed the presence of functional groups and chemical bonding in PLA–CA–SiO_2_ composite membranes. Composite membranes have shown good UV–A, UV–B and visible radiation blocking efficiency. Additionally, radiation blockage increased with higher concentrations of silica nanoparticles. Thermal infrared imaging showed effectiveness as protective textiles under direct sun radiation. The concentration of silica also affects wettability and resistance to hydrostatic pressure. The S3 composite membrane demonstrated a water contact angle of 143.7° and a hydrostatic pressure resistance of 27 kPa. The microporous structure of the composite membranes improved breathability and enhanced air and vapor transport. Tensile testing revealed that the small concentrations of silica nanoparticles enhanced the tensile strength and toughness of membranes. The S1 composite membrane demonstrated a tensile strength of 11.2 MPa, a Young’s modulus of 329 MPa, and an elongation at break of 98.5%. The developed biopolymeric composite membranes demonstrated desirable properties typical of electrospun composites and showed strong potential to replace petroleum-based polymers in various applications.

## Figures and Tables

**Table 1:** PLA–CA–SiO_2_ composite membranes codes and silicon dioxide concentrations.

Electrospun Composite Membrane Code	PLA and CA Concentration (*v*/*v*)	SiO_2_ Concentration (*w*/*w*)%
S0		0
S1		0.5
S2	PLA 95–CA 5	1.5
S3		3
S4		5
S5		7

**Table 2:** Thermal properties i.e., $T_{on-set}$, $T_{50\%\;WL}$, $T_{d-max}$, $T_{g}$, and $T_{m}$ of PLA0–CA–SiO_2_ composite membranes.

Specimen	$T_{on-set}$ (°C)	$T_{50\%\;WL}$ (°C)	$T_{d-max}$ (°C)	$T_{g}$ (°C)	$T_{m}$ (°C)
S0	298.9	322.3	329.6	61.3	144.2
S1	308.1	331.8	337.6	59.3	153.1
S2	313.6	337.1	345.6	59.2	153.5
S3	285.9	318.9	328.9	59.3	151.4
S4	287.9	322.7	327.9	59.2	151.8
S5	284.6	320.8	325.8	58.8	151.9

## Data Availability

The original contributions presented in this study are included in the article. Further inquiries can be directed to the corresponding author.
